# Pet Ownership and Longitudinal Change in Physical Function: Evidence From the BLSA

**DOI:** 10.1093/geroni/igab046.830

**Published:** 2021-12-17

**Authors:** Erika Friedmann, Nancy Gee, Eleanor Simonsick, Erik Barr, Barbara Resnick, Emily Werthman, Ikmat Adesanya

**Affiliations:** 1 University of Maryland, Baltimore, Maryland, United States; 2 Virginia Commonwealth University, Richmond, Virginia, United States; 3 National Instute on Aging/NIH, Baltimore, Maryland, United States; 4 University of Maryland Baltimore, University of Maryland Baltimore, Maryland, United States; 5 University of Maryland School of Nursing, Baltimore, Maryland, United States; 6 University of Maryland, Baltimore, Baltimore, Maryland, United States

## Abstract

Successful aging depends on avoiding disease and disability, maintaining high physical and cognitive function, and psychological adaptation. Research examining the relationship of pet ownership (PO) or human-animal interaction (HAI) to human health supports contributions to these successful ag-ing-related outcomes at some point in the life-cycle, mostly in populations with diseases or disabili-ties. We examine the contributions of PO to maintaining physical capacity among generally healthy community-dwelling older participants in the Baltimore Longitudinal Study of Aging (BLSA). Partici-pants’ [N=637, mean age=68.3 years (SD=9.6), pet owners N=149] completed a standardized physi-cal function test battery (among other measures) every 1-4 years and a ten-year PO history. Linear mixed, or generalized linear mixed, models with time varying PO were used to examine change in successful aging-related outcomes over up to 13 years [mean=7.5, (SD=3.6)] according to PO. Physi-cal function declined across all domains examined, but was observed to be less severe with PO in overall physical performance (p<0.001), rapid gait speed (p=0.041), 400-meter walk time (p<0.001), and reported physical wellbeing (p=0.032). No differences were observed for grip strength (p=0.56), usual gait speed (p=0.07), and leisure time physical activity (p=0.26) after con-trolling for age. This study provides the first longitudinal evidence that PO may promote successful aging among community-dwelling healthy older adults by moderating age-related declines in physical functional status in late-life.

